# Five-year clinical outcomes in patients with diabetes mellitus treated with polymer-free sirolimus- and probucol-eluting stents versus second-generation zotarolimus-eluting stents: a subgroup analysis of a randomized controlled trial

**DOI:** 10.1186/s12933-016-0429-y

**Published:** 2016-09-01

**Authors:** Yukinori Harada, Roisin Colleran, Sebastian Kufner, Daniele Giacoppo, Tobias Rheude, Jonathan Michel, Salvatore Cassese, Tareq Ibrahim, Karl-Ludwig Laugwitz, Adnan Kastrati, Robert A. Byrne, for the Intracoronary Stenting and Angiographic Results: Test Efficacy of Sirolimus- and Probucol- and Zotarolimus- Eluting Stents (ISAR-TEST 5) Investigators

**Affiliations:** 1 Deutsches Herzzentrum München, Technische Universität München, Lazarettstraße 36, Munich, Germany; 2 1. medizinische Klinik, Klinikum rechts der Isar, Technische Universität München, Ismaninger Straße 22, Munich, Germany

**Keywords:** Diabetes, Drug-Eluting Stent, Probucol, Sirolimus, Zotarolimus

## Abstract

**Background:**

Improved outcomes in patients with diabetes mellitus undergoing percutaneous coronary intervention remain an unmet clinical need. We assessed the long-term efficacy and safety of novel polymer-free sirolimus- and probucol-eluting stent in diabetic patients enrolled in intracoronary stenting and angiographic results: test efficacy of sirolimus- and probucol-eluting versus zotarolimus-eluting stents 5 trial.

**Methods:**

In a pre-specified subgroup analysis, outcomes of diabetic patients treated with a sirolimus- and probucol-eluting stent or a second-generation zotarolimus-eluting stent were compared. The primary endpoint was a device-oriented composite outcome comprising cardiac death, target vessel-related myocardial infarction (MI), or target lesion revascularization (TLR) at 5-year follow-up. Event-free survival was assessed using the Kaplan–Meier method. Hazard ratios (HR) and 95 % confidence intervals (CI) were estimated from univariate Cox proportional hazards models.

**Results:**

A total of 870 patients with diabetes mellitus were treated with either a sirolimus- and probucol-eluting stent (n = 575) or a second-generation zotarolimus-eluting stent (n = 295). At 5 years, the rate of device-oriented composite endpoint was comparable between the sirolimus- and probucol-eluting stent and the second-generation zotarolimus-eluting stent (32.9 versus 33.4 %, HR 0.88, 95 % CI 0.76–1.26). No significant differences were observed between the sirolimus- and probucol-eluting stent and the second-generation zotarolimus-eluting stent groups in the incidence of cardiac death (15.6 versus 16.7 % HR 0.92, 95 % CI 0.63–1.32), target-vessel MI (4.6 versus 6.6 %, HR 0.73, 95 % CI 0.40–1.34), and TLR (18.6 versus 18.8 %, HR 1.00, 95 % CI, 0.72–1.41). The rate of definite or probable stent thrombosis was low and similar in both groups (2.5 versus 2.6 %, HR 1.02, 95 % CI, 0.41–2.52).

**Conclusions:**

In patients with diabetes the long-term efficacy and safety of a polymer-free sirolimus- and probucol-eluting stent were comparable to a second-generation durable polymer zotarolimus-eluting stent.

*Trial registration* ClinicalTrials.gov NCT00598533. Registered 10 January 2008

## Background

High rates of adverse events remain a cause of concern in patients with diabetes mellitus after percutaneous coronary intervention for obstructive coronary artery disease [1, 2]. Second-generation durable polymer drug-eluting stents (DES), including a durable polymer zotarolimus-eluting stent, have demonstrated good efficacy in the treatment of coronary artery disease in diabetic patients [3, 4]. However, persistent inflammatory response to durable polymer coatings of DES is recognized as a leading cause of delayed arterial healing and one of the key factors underlying late stent failure in patients who undergo DES implantation [5, 6]. Indeed, this issue is of particular relevance in the setting of diabetic patients with high atherothrombotic risk [2, 7].

Against this background polymer-free DES showed non-inferior short-term angiographic outcomes in comparison with a durable polymer-based everolimus-eluting stent in the setting of diabetic patients [8]. However, the efficacy and safety of polymer-free DES compared with durable polymer-based second-generation DES in diabetic patients are poorly defined and very long-term outcomes are underexplored with data almost exclusively deriving from comparison with outdated first-generation polymer-based DES [9].

The intracoronary stenting and angiographic results: test efficacy of sirolimus- and probucol-eluting versus zotarolimus-eluting stents (ISAR-TEST) 5 trial demonstrated the non-inferiority of a polymer-free dual drug sirolimus- and probucol-eluting stent in comparison with a durable polymer DES for the clinical endpoints of interest at 1 year follow up [10]. In addition, long-term follow-up of patients enrolled in this trial showed consistent results over time with comparable safety and efficacy between the two devices at 5 years follow up [11]. The aim of our study was to assess 5-year clinical outcomes of patients with diabetes mellitus who underwent angioplasty with a polymer-free dual drug sirolimus- and probucol-eluting stent or a second-generation durable polymer zotarolimus-eluting stent implantation enrolled in the ISAR-TEST 5 trial.

## Methods

### Study population and study protocol

The primary analysis of the ISAR-TEST 5 clinical trial was previously reported [10]. Patients with diabetes mellitus represented a pre-specified subgroup of interest according to the trial protocol. In brief, the ISAR-TEST 5 trial was a randomized, non-inferiority trial conducted between February 2008 and August 2009, in which 3002 patients with ischemic symptoms or evidence of inducible or spontaneous myocardial ischemia and one or more ≥50 % de novo stenosis located in native coronary vessels were assigned in a 2:1 treatment allocation to receive either a polymer-free dual drug sirolimus- and probucol-eluting stent (backbone Yukon stent, Translumina, Hechingen, Germany; stent currently commercially available as Coroflex Isar stent, B. Braun Melsungen AG, Melsungen, Germany) or a second-generation durable polymer zotarolimus-eluting stent (Endeavor Resolute, Medtronic Vascular, Santa Rosa, Ca, USA). Minimal exclusion criteria were applied, with exclusion only of patients presenting left main stenosis, cardiogenic shock, malignancies, life expectancy <12 months, known allergies to study medication and pregnancy. Randomization sequence was computer generated with allocation concealment by means of sealed opaque envelopes. Randomization was done immediately after crossing the lesion with the guide-wire. In case of multiple lesions requiring intervention, the same assigned stent was to be implanted in all lesions.

The trial was registered at http://www.clinicaltrials.gov. (trial identifier: NCT00598533). The study was conducted in accordance with the provisions of the Declaration of Helsinki and with the International Conference on Harmonization Good Clinical Practices. The trial protocol was approved by the institutional ethics committee of the participating centers and all included patients provided specific written informed consent.

Patients were systematically evaluated at 1, 12, 24 and 60 months by telephone call or office visit. All events were adjudicated and classified by an event adjudication committee unaware of treatment allocation. According to the trial protocol, follow-up coronary angiography was scheduled at 6–8 months and both off-line quantitative coronary angiography and qualitative assessment were performed in an independent core laboratory (ISARESEARCH Center, Munich, Germany).

### Study devices and anti-thrombotic medications

The polymer-free stent platform consists of a pre-mounted, thin strut (87 µm), sand-blasted, 316L stainless steel microporous stent coated with a matrix of sirolimus (0.7 %) and probucol (0.7 %) [10, 12, 13]. The distribution concentration of sirolimus is 120 mg/cm^2^ stent and of probucol is 100 mg/cm^2^. Non-clinical testing showed that no traces of sirolimus or probucol are observable beyond 6–8 weeks [13]. The second-generation durable polymer zotarolimus-eluting stent uses a bare metal cobalt-chromium platform coated with zotarolimus and the BioLinx polymer system [14].

An oral loading dose of 600-mg clopidogrel was administered to all patients at least 2 h before the intervention, regardless of whether the patient was taking clopidogrel before being admitted. During the procedure, intravenous aspirin, heparin or bivalirudin, and glycoprotein IIb/IIIa inhibitor administration was at the discretion of the operators. After the intervention, all patients, regardless of treatment allocation, were prescribed 200 mg/day aspirin indefinitely, clopidogrel 150 mg for the first 3 days or until discharge followed by 75 mg/day for at least 6 months.

### End points and definitions

The primary endpoint of ISAR-TEST 5 was the device-oriented composite of cardiac death, target vessel-related myocardial infarction, or target lesion revascularization at 5 years (final study follow up). Secondary endpoints were as follows: cardiac death, all-cause death, target vessel myocardial infarction, any myocardial infarction, target lesion revascularization, any revascularization, target vessel revascularization and the incidence of definite/probable stent thrombosis (by Academic Research Consortium definition) in the diabetic subgroup at 5 years. Detailed definitions of endpoints have been previously reported [10].

### Statistical analysis

The analysis of primary and secondary endpoints was planned to be performed on an intention-to-treat basis. Continuous data are presented as mean (standard deviation) or median (25th–75th percentiles) according to data distribution testing by the Kolmogorov–Smirnov test. Categorical data are presented as counts and proportions (%). Differences of clinical characteristics between groups were inspected using the Student’s *t* or Wilcoxon rank sum test for continuous variables and the Chi squared or Fisher’s exact test for categorical variables (patient-level). Angiographic differences between groups were compared using generalized estimating equations for non-normally distributed data [15], in order to address intra-patient correlation in patients who underwent multi-lesion intervention. Event-free survival was assessed using the Kaplan–Meier method and differences were quantified by the log-rank test. Hazard ratios (HR) with accompanying 95 % confidence intervals (CI) were estimated from univariate Cox proportional hazards models. The proportional hazards assumption checked by the method of Grambsch and Therneau [16] was fulfilled in all cases in which we used Cox proportional hazards models. Statistical software S-PLUS, version 4.5 (S-PLUS, Insightful Corp, Seattle, Wa, USA) was used for analyses.

## Results

### Patient, lesion, and procedural characteristics and angiographic outcomes

**Fig. 1:**
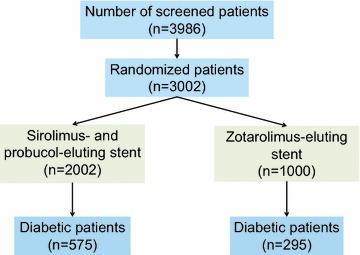
Patient flow in this study

Of a total of 3002 patients enrolled in the ISAR-TEST 5 trial, 870 patients with diabetes mellitus were identified: 575 patients were assigned to treatment with polymer-free sirolimus- and probucol-eluting stent and 295 to durable polymer zotarolimus-eluting stent (Fig. 1). The groups were well matched in terms of baseline patient and lesion characteristics (Table 1). Prevalence of patients requiring insulin treatment (P = 0.43) and oral anti-diabetic therapy only (P = 0.94) were comparable between groups. A total of 1288 coronary lesions received percutaneous coronary intervention (sirolimus- and probucol-eluting stent, n = 849; zotarolimus-eluting stent, n = 439). Prevalence of patients with multiple lesions which treated with stent implantation was comparable between groups (P = 0.61). Procedural characteristics were broadly comparable between the two groups except post-procedural minimal luminal diameter and percent diameter stenosis (Table 2).

**Table 1:** Baseline clinical characteristics

Patient-level characteristics	Sirolimus- and probucol-eluting stent (n = 575)	Zotarolimus-eluting stent (n = 295)	P value
Age (years)	69 (61–76)	70 (62–76)	0.40
Female	150 (26.1)	79 (26.8)	0.83
*Diabetes mellitus therapy*			
Insulin	197 (34.0)	109 (37.0)	0.43
Oral antidiabetic drugs	289 (50.0)	149 (51.0)	0.94
Hypertension	547 (95.1)	281 (95.3)	0.94
Hypercholesterolemia	389 (68.0)	188 (64.0)	0.25
Current smoker	105 (18.0)	52 (18.0)	0.82
Prior myocardial infarction	177 (30.8)	85 (28.8)	0.55
Prior bypass surgery	59 (10.3)	34 (11.5)	0.57
*Clinical presentation*			0.46
Silent ischemia	36 (6.3)	15 (5.1)	
Stable angina	324 (56.3)	154 (52.2)	
Unstable angina	98 (17.0)	61 (20.7)	
Non ST-segment elevation myocardial infarction	73 (12.7)	45 (15.3)	
ST-segment elevation myocardial infarction	44 (7.7 %)	20 (6.8 %)	
Multi-vessel disease	517 (89.9)	263 (89.2)	0.73
Ejection fraction (%)^a^	54 (44-60)	55 (41–61)	0.56

Lesion-level characteristics	Sirolimus- and probucol-eluting stent (n = 849)	Zotarolimus-eluting stent (n = 439)	P value
*Target vessel*			0.11
Left anterior descending	336 (39.6)	196 (44.6)	
Left circumflex	236 (27.8)	123 (28.0)	
Right coronary artery	277 (32.6)	120 (27.3)	
Chronic total occlusion	56 (6.6)	22 (5.0)	0.26
Bifurcation	200 (23.6)	112 (25.5)	0.44
Ostial	152 (17.9)	80 (18.2)	0.89
Complex morphology	626 (74.0)	336 (77.0)	0.27
Lesion length (mm)	16.6 ± 9.5	17.9 ± 10.4	0.07
Reference vessel diameter (mm)	2.75 ± 0.52	2.79 ± 0.51	0.36
Minimal luminal diameter (mm)	0.91 ± 0.50	0.92 ± 0.47	0.83
Percent diameter stenosis (%)	67 ± 16	67 ± 15	0.83

Data shown as mean ± SD, median (25th–75th percentiles), or n (%)

^a^Data available for 725 patients (86.7 %)

**Table 2:** Procedural characteristics and angiographic outcomes

Lesion-level characteristics	Sirolimus- and probucol-eluting stent (n = 849)	Zotarolimus-eluting stent (n = 439)	P value
Balloon diameter (mm)	3.05 (2.59–3.47)	3.02 (2.60–3.45)	0.82
Stented length (mm)	25 (18–34)	24 (18–33)	0.23
*In stent analysis*			
Post-procedural minimal luminal diameter (mm)	2.50 ± 0.50	2.57 ± 0.49	0.045
Post-procedural percent diameter stenosis (%)	12 ± 7	11 ± 7	0.03
At follow-up minimal luminal diameter (mm)^a^	2.13 ± 0.73	2.20 ± 0.72	0.18
At follow-up percent diameter stenosis (%)^a^	24 ± 22	23 ± 21	0.68
Late lumen loss (mm)^a^	0.36 ± 0.63	0.36 ± 0.59	0.48
*In segment analysis*			
Post-procedural minimal luminal diameter (mm)	2.23 ± 0.58	2.26 ± 0.55	0.33
Post-procedural percent diameter stenosis (%)	22 ± 12	22 ± 12	0.83
At follow-up minimal luminal diameter (mm)^a^	1.90 ± 0.70	1.98 ± 0.69	0.10
At follow-up percent diameter stenosis (%)^a^	33 ± 20	32 ± 19	0.20
Late lumen loss (mm)^a^	0.31 ± 0.61	0.26 ± 0.57	0.34
Binary restenosis^a^	107 (17.0)	57 (17.2)	0.95

Data shown as mean ± SD or median (25th–75th percentiles) or n (%)

^a^Data available for 961 lesions (74.6 %)

Angiographic outcomes at 6–8 months are shown in Table 2. In-stent late lumen loss and in-segment percent diameter stenosis at angiographic follow-up were not different between the treatment groups.

### Outcomes at 5 years

**Fig. 2:**
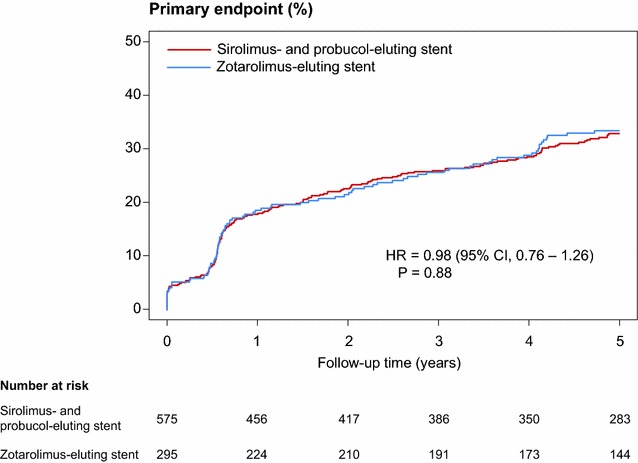
Time to event curve in cumulative incidences of primary endpoint. Primary endpoint is the device-oriented composite of cardiac death, target vessel-related myocardial infarction, or target lesion revascularization. Hazard ratios and P values are derived from Cox proportional hazard methods. *CI* confidence interval, *HR* hazard ratio

**Fig. 3:**
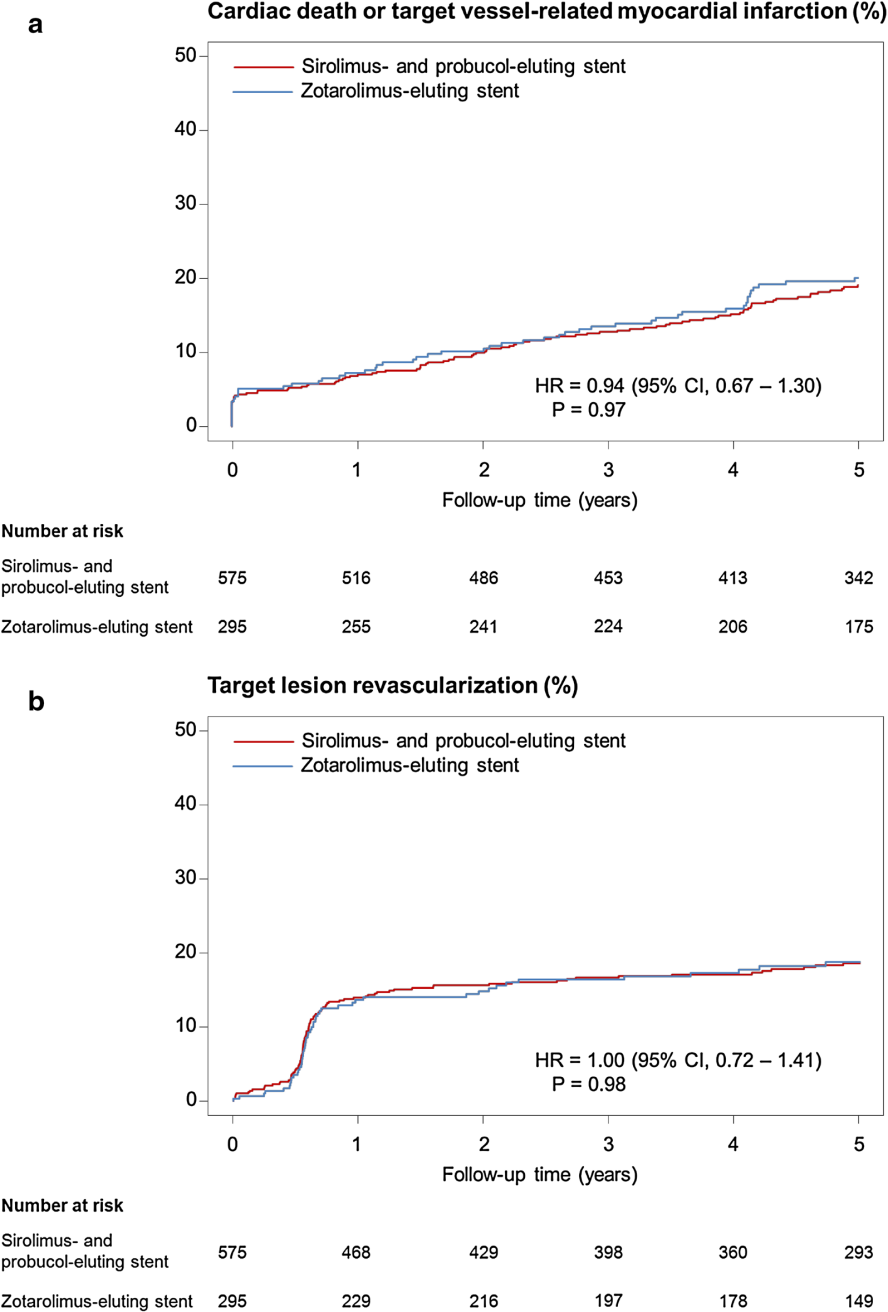
Time to event curve in cumulative incidences of the components of primary endpoint (**a**) the composite of cardiac death, target vessel-related myocardial infarction. **b** Target lesion revascularization. Hazard ratios and P values are derived from Cox proportional hazard methods. *CI* confidence interval, *HR* hazard ratio

Five-year clinical outcomes are shown in Table 3. There was no significant difference in the occurrence of the primary composite endpoint at 5 years between the polymer-free sirolimus- and probucol-eluting stent and durable polymer zotarolimus-eluting stent groups (32.9 versus 33.4 % respectively, HR = 0.88, 95 % CI, 0.76–1.26; P = 0.88)(Fig. 2). In terms of the individual components of the primary endpoint, rates were similar between the two groups: the composite of cardiac death or target vessel myocardial infarction was, 19.1 versus 20.1 % respectively (HR 0.94, 95 % CI, 0.67–1.30; P = 0.70) (Fig. 3a), and target lesion revascularization was, 18.6 versus 18.8 % respectively (HR 1.00, 95 % CI, 0.72–1.41; P = 0.98) (Fig. 3b). There were also no differences of the incidences of other secondary endpoints of all-cause death, any myocardial infarction, any revascularization, and target vessel revascularization.

**Table 3:** Clinical results at 5 years

	Sirolimus- and probucol-eluting stent (n = 575)	Zotarolimus-eluting stent (n = 295)	Hazard ratio [95 % CI]	P value
Cardiac death, target vessel-related myocardial infarction or target lesion revascularization	178 (32.9)	91 (33.4)	0.98 [0.76–1.26]	0.88
Cardiac death or target vessel-related myocardial infarction	101 (19.1)	54 (20.1)	0.94 [0.67–1.30]	0.70
Cardiac death	81 (15.6)	44 (16.7)	0.92 [0.63–1.32]	0.64
Target vessel-related myocardial infarction	26 (4.6)	18 (6.6)	0.73 [0.40–1.34]	0.31
Target lesion revascularization	100 (18.6)	50 (18.8)	1.00 [0.72–1.41]	0.98
All-cause death	133 (24.4)	79 (27.8)	0.84 [0.63–1.11]	0.21
Any myocardial infarction	37 (6.5)	21 (7.6)	0.90 [0.53–1.54]	0.70
Any revascularization	234 (43.4)	124 (46.9)	0.92 [0.74–1.15]	0.48
Target vessel revascularization	144 (26.8)	70 (26.2)	0.82 [0.78–1.37]	0.82
Definite or probable stent thrombosis	14 (2.5)	7 (2.6)	1.02 [0.41–2.52]	0.97
Definite stent thrombosis	7 (1.2)	4 (1.6)	0.89 [0.26–3.04]	0.85
Probable stent thrombosis	7 (1.2)	3 (1.0)	1.19 [0.31–4.60]	0.80

Data shown as n (%) or hazard ratio [95 % CI]

Rates are estimated by Kaplan–Meier method; hazard ratios and p values were calculated by Cox’s proportional hazard methods

*CI* confidence intervals

**Fig. 4:**
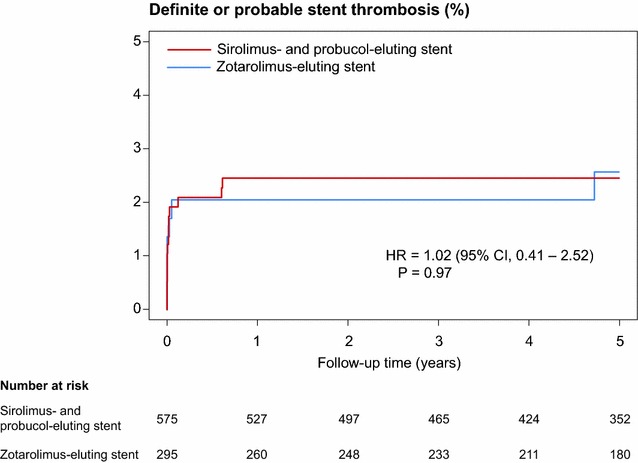
Time to event curve in cumulative incidences of the definite or probable stent thrombosis. Hazard ratios and P values are derived from Cox proportional hazard methods

The rate of definite or probable stent thrombosis was low and similar in both groups (2.5 versus 2.6 % respectively, HR 1.02, 95 % CI, 0.41–2.52; P = 0.97), with only one case occurring in the durable polymer zotarolimus-eluting stent group after 12 months (Fig. 4).

## Discussion

### Main findings in this study

In the high-risk subgroup of patients with diabetes mellitus enrolled in a large-scale randomized trial with broad inclusion criteria, the composite primary endpoint of cardiac death, myocardial infarction-related to the target vessel, or target lesion revascularization at 5-year follow-up in subjects treated with a polymer-free sirolimus- and probucol-eluting stent was non-inferior compared to that in patients treated with a second-generation durable polymer zotarolimus-eluting stent. Moreover, each of the individual cardiovascular endpoints was comparable between groups. In particular, the incidence of stent thrombosis was low and comparable in both treatment groups with few events beyond 12 months.

### Relationship between polymer coatings and adverse cardiac events

In patients with diabetes mellitus undergoing coronary stenting, the rates of adverse cardiac events such as in-stent restenosis, myocardial infarction and cardiac death remain high even after implantation of second-generation DES [1–3]. Moreover, these adverse cardiac events rates are higher in diabetic patients who require insulin treatment than in those who do not require it [17]. Indeed, higher incidences of in-stent neoatherosclerosis and very late stent thrombosis were observed in diabetic patients compared with non-diabetic patients [18, 19]. Both of the latter processes are likely related to delayed arterial healing [20, 21], which is at least in part caused by an inflammatory reaction to the polymer coatings used on DES [5, 6]. On this basis, polymer-free stent design might be an approach particularly well-suited for the treatment of coronary artery disease in diabetic patients.

### Efficacy and safety of polymer-free drug-eluting stents

Prior investigation in diabetic patients showed that a polymer-free sirolimus-eluting stent had similar long-term efficacy and safety compared with a first-generation paclitaxel-eluting stent [9]. However, this data was limited by the fact that the comparator stent was an early-generation DES with only moderate antirestenotic efficacy, which has subsequently fallen out of clinical use. In addition, the study stent was coated only with sirolimus, an approach that likely does not result in adequate clinical efficacy. In our study, in a large cohort of diabetic patient we showed comparable clinical efficacy at 5 years between a polymer-free sirolimus- and probucol-eluting stent compared with a high performance second-generation durable polymer zotarolimus-eluting stent. As previously reported the improved angiographic antirestenotic efficacy with the polymer-free stent used in our study (in comparison with a similar single-drug polymer-free stent) are likely due to the incorporation of probucol in the stent coating [13, 22]. This compensates for the inherently less favorable drug-release kinetic seen with polymer-free DES. The mechanism of benefit is likely twofold: as probucol is highly lipophilic it can retard the release of sirolimus from the stent surface and improve tissue drug levels, in addition, due to the antioxidant effects of probucol it targets a separate component of the restenotic response cascade [23, 24].

Importantly, the polymer-free sirolimus- and probucol-eluting stent also demonstrated a low incidence of stent thrombosis out to 5 years. Indeed we observed no cases of stent thrombosis beyond 1 year in patients treated with the polymer-free stent in this study. On the other hand, it should be observed that no clear advantage could be seen with polymer-free stents in comparison to durable polymer stents with regard to very late stent thrombosis. Indeed this is broadly in line with results of a recent meta-analysis of both diabetic and nondiabetic patients [25], though this lack of difference must be interpreted in light of the low event rates in both groups.

With regard to angiographic antirestenotic efficacy, the late lumen loss observed in patients with diabetes in our study was 0.36 mm (in stent) both with sirolimus- and probucol-eluting and zotarolimus-eluting stents. This is somewhat higher than in other studies investigating patients with diabetes. For example in the SPIRIT V diabetic study, late loss was 0.19 mm; in the RESORVOIR study, 0.24 mm with everolimus-eluting stent [8, 26]. Although the reasons for this are unclear, this may be related to baseline patient and lesion complexity: the inclusion criteria in our study were broader and exclusion criteria were fewer. Moreover, our results may be expected to be representative of real-world practice and therefore broadly applicable to diabetic patients in a wide variety of settings.

## Limitations

Our study has several limitations. First, because this is a post hoc analysis at 5 years, results must be interpreted with caution. Second, despite including 870 patients, this substudy was not powered to show a difference in the occurrence of the primary endpoint between the two groups at 5 years as well as in rarely-occurring individual endpoints such as stent thrombosis. Third, although the target lesion revascularization rates of both stents in our study were relatively high, these results were likely influenced by requirement for protocol-mandated angiographic follow-up. This is known to inflate the rate of revascularization in comparison with routine clinical practice. In fact, most cases with target lesion revascularization were observed between 6 and 8 months after index intervention. However, despite potential for greater absolute differences between stents in trials with angiographic follow-up, relative differences observed are expected to be real [27]. Fourth, although we recommended patients to continue dual antiplatelet therapy at least 12 months, data concerning the actual duration of dual anti-platelet therapy received was not captured.

## Conclusions

In the setting of a large-scale randomized trial with broad inclusion criteria, cardiovascular outcomes at 5 years follow-up of diabetic patients treated with a polymer-free sirolimus- and probucol-eluting stent were non-inferior to those of patients receiving a second-generation durable polymer zotarolimus-eluting stent. Rates of stent thrombosis were comparable and satisfactory low, with few events beyond 12 months.

## Abbreviations

CI: confidence intervals
DES: drug-eluting stent
HR: hazard ratios
ISAR-TEST: intracoronary stenting and angiographic resultstest efficacy of sirolimus- and probucol-eluting versus zotarolimus-eluting stents
